# Polysubstance Use in Cannabis Users Referred for Treatment: Drug Use Profiles, Psychiatric Comorbidity and Cannabis-Related Beliefs

**DOI:** 10.3389/fpsyt.2013.00079

**Published:** 2013-08-07

**Authors:** Jason P. Connor, Matthew J. Gullo, Gary Chan, Ross McD. Young, Wayne D. Hall, Gerald F. X. Feeney

**Affiliations:** ^1^Alcohol and Drug Assessment Unit, Princess Alexandra Hospital, Brisbane, QLD, Australia; ^2^Faculty of Health Sciences, Centre for Youth Substance Abuse Research, The University of Queensland, Brisbane, QLD, Australia; ^3^Discipline of Psychiatry, School of Medicine, The University of Queensland, Brisbane, QLD, Australia; ^4^Faculty of Health, Queensland University of Technology, Brisbane, QLD, Australia; ^5^The University Queensland Centre for Clinical Research, The University of Queensland, Brisbane, QLD, Australia

**Keywords:** cannabis, latent class, drugs, comorbidity, expectancy, self-efficacy, treatment seeking

## Abstract

**Background:** Population-based surveys demonstrate cannabis users are more likely to use both illicit and licit substances, compared with non-cannabis users. Few studies have examined the substance use profiles of cannabis users referred for treatment. Co-existing mental health symptoms and underlying cannabis-related beliefs associated with these profiles remains unexplored.

**Methods:** Comprehensive drug use and dependence severity (Severity of Dependence Scale-Cannabis) data were collected on a sample of 826 cannabis users referred for treatment. Patients completed the General Health Questionnaire, Cannabis Expectancy Questionnaire, Cannabis Refusal Self-Efficacy Questionnaire, and Positive Symptoms and Manic-Excitement subscales of the Brief Psychiatric Rating Scale. Latent class analysis was performed on last month use of drugs to identify patterns of multiple drug use. Mental health comorbidity and cannabis beliefs were examined by identified drug use pattern.

**Results:** A three-class solution provided the best fit to the data: (1) cannabis and tobacco users (*n* = 176), (2) cannabis, tobacco, and alcohol users (*n* = 498), and (3) wide-ranging substance users (*n* = 132). Wide-ranging substance users (3) reported higher levels of cannabis dependence severity, negative cannabis expectancies, lower opportunistic, and emotional relief self-efficacy, higher levels of depression and anxiety and higher manic-excitement and positive psychotic symptoms.

**Conclusion:** In a sample of cannabis users referred for treatment, wide-ranging substance use was associated with elevated risk on measures of cannabis dependence, co-morbid psychopathology, and dysfunctional cannabis cognitions. These findings have implications for cognitive-behavioral assessment and treatment.

## Introduction

Between 2.8 and 4.5% of the world’s adult population have used cannabis in the past year (1), making it globally the most widely used illicit substance. General population estimates indicate that up to 1.3% are cannabis dependent (2). Individuals who use cannabis are also more likely to use other illicit substances (3). The association between cannabis use and mental health problems is well documented (4, 5). Analyses of cannabis users in population-based surveys have identified substance use ‘typologies’ though latent class modeling [e.g., (6)]. These typologies can inform public health and targeted prevention approaches. The ‘typology’ of cannabis users referred for treatment is likely to differ from that in the general population. Polysubstance use, mental health comorbidity and underlying acquired cannabis-related beliefs associated with these substance use profiles require further investigation.

Latent Class Analysis (LCA) has been widely applied in population-based alcohol and drug research to estimate probability of substance use sub-classes, or ‘typologies.’ Most generate class solutions that include: (a) no or limited substance use, (b) moderate substance use, and (c) wide-ranging substance use. In addition to varying range of substances captured across studies, the final number of class solutions and prevalence rates per solution varies as a function of the population sampled and period of drug use captured (typically lifetime or past 12 month use). For example, a representative sample from the British National Household Survey (*n* = 8538, mean age 42.55 years) generated a three-class solution of 12 month illicit drug use: (1) no polydrug use (95.78%), (2) moderate polydrug use (3.44%), and (3) wide-ranging polydrug use (0.77%) (3). Based on lifetime illicit substance use data from an Australian Twin Study (*n* = 6265, mean age 30 years), Lynskey et al. (7) identified a 5-class model: (1) low use (68.5%), (2) moderate use of all substances (17.8%), (3) high use of stimulants and hallucinogens and low use sedatives and opioids (6.6%), (4) high use sedatives and opioids and low use of stimulants and hallucinogens (3.0%), and (5) uniformly high use across all substances (4.2%).

Examining lifetime use of all substances (illicit and licit) of younger age groups from the Australian National Drug Strategy Household Survey (*n* = 1402, 12–17 years), White et al. (8) found a three-class model that included: (1) alcohol only (79.6%), (2) limited range multidrug users (18.3%), and extended range multidrug users (2%). In community-based samples of cannabis users the percentage of wide-ranging substance use increases to 21% (past 3 months) (9). The prevalence rates of de Dios et al.’s (9) other two LCA cannabis classes were Unaffected/Mild Users (37%) and Moderate Problem Users (42%).

Comparisons between studies are difficult because narrower substance use time frames reduce prevalence rates. Targeting specific substance using populations increases the prevalence of polysubstance use. Broader timeframes (e.g., lifetime use) are less reliable in reporting recent polysubstance use patterns. Narrower assessment timeframes represent more clinically relevant data, but can lack power because of the low prevalence of use of some substances.

Mental health problems often co-occur with substance use disorders, including psychotic-like symptoms (10–12). Substance use LCA studies permit a more precise investigation of patterns of psychiatric comorbidity. Wider-ranging LCA substance use classes have previously been associated with elevated psychological distress (8), increased mood and anxiety problems, suicide attempts (3, 7) and treatment seeking (3). When alcohol dependent subjects are examined within population-based surveys, those classified by LCA as having a high probability of heavy alcohol consumption as well as heavy illicit drug use, are more likely to have co-existing generalized anxiety and major depressive disorders (13). Studies that have extracted cannabis users from nationally representative data sets, observe similar deficits in functioning within those classes reporting higher risk for multiple substance use (6). These findings suggest psychiatric severity increases linearly with increased polysubstance use.

No LCA studies have examined the substance use profiles and accompanying mental health comorbidity of individuals referred for cannabis use treatment. These profiles are likely to be different from population-based studies. To improve assessment and treatment of this group, it is also of benefit to extend beyond broader mental health functioning measures and examine additional etiological factors, especially cannabis-related beliefs which can serve as targets for evidence-based psychological interventions. Two such targets for cognitive-behavioral treatment are outcome expectancies and substance-refusal self-efficacy. Both carry strong Social Cognitive Theory (SCT) pedigrees (14–16).

Outcome expectancies are sometimes referred to as ‘if … then’ statements that reflect the perceived behavioral and affective consequences of engaging in specific behaviors (17). Cannabis expectancy scales are typically represented by two higher-order expectancy factors, representing positive (e.g., “I have more self-confidence when smoking cannabis”) and negative (e.g., “Smoking cannabis makes me confused”) expectancies [see (18)]. Self-efficacy refers to a person’s belief they can successfully or unsuccessfully regulate their behavior (e.g., “I am very sure I could not resist smoking cannabis when I feel upset”). Cannabis refusal self-efficacy is considered a central psychological mechanism that predicts post-treatment consumption (19) and abstinence (20, 21). Expectancy ‘challenges’ have been applied in alcohol use prevention and treatment (22), but progress in cannabis has been hampered by a lack of cannabis-specific assessment tools. Cannabis expectancy and refusal self-efficacy scales have recently been validated for use in clinical populations (18, 23).

Polysubstance use varies widely in definition. Here we define it as two or more substances used in the past month. In this study of cannabis users referred for treatment, we predicted a continuum of past month polysubstance use that would range from cannabis only (and no/low licit drug use) to wide-ranging polysubstance use. We make no *a priori* assumptions about the number of LCA solutions, but predicted a higher prevalence of polysubstance use within wider-ranging profiles compared to community [e.g., (9)] and general population [e.g., (3)] samples. Mental health functioning should be poorer across all class solutions, when compared to community population norms. Consistent with the findings of Lynskey et al. (7) and Smith et al. (3), symptoms of mood and anxiety disorders are likely to be more impaired in users with wider-ranging drug profiles. Psychotic-like symptoms, on the other hand, are likely to show a dose-response relationship, such that the classes with more severe cannabis dependence will display a higher symptom severity (11). Patients who use cannabis with no or limited other substance use are expected to have greater opportunities to form more salient cannabis-related beliefs (18), and should have higher cannabis expectancy and lower cannabis refusal self-efficacy.

The main aim of this study is to identify polysubstance typologies for cannabis users in treatment. Based on these typologies, it is expected that additional information on associated mental health functioning and cognitive treatment targets will assist researchers and health practitioners provide more effective assessment approaches. These assessments are likely to result in more tailored interventions for this group.

## Materials and Methods

### Participants

The sample comprised 827 individuals who were referred for assessment as part of the Queensland Illicit Drug Diversion Initiative (QIDDI). The program involves a 2-h comprehensive assessment of substance use and psychosocial functioning that incorporates motivational interviewing (MI). Where indicated, referral for further treatment is provided. Of the 827 participants, 623 (77.2%) were men, and the mean age was 25.46 years (SD = 8.35). The majority were born in Australia (692; 83.7%) or New Zealand (53; 6.4%), and 49 (5.9%) identified themselves as Indigenous Australians. Almost half (46.4%) scored above the Severity of Dependence Scale-Cannabis (SDS-C) screening cut-off for cannabis dependence [≥3, (24)]. Average weekly cannabis consumption was 3.54 (SD = 4.90) g and the average SDS-C score was 3.13 (SD = 3.20). Past month alcohol and other drug use is presented in Table 1. The 4-week window was chosen to better reflect current polysubstance use. Previous studies reporting 12 month or lifetime use have less clinical utility (e.g., a patient who used cannabis once and alcohol once would fit criteria of a polysubstance user in lifetime studies). Of the original sample, 20 participants (2.4%) were excluded from the main analysis due to missing values on one or more drug-related variables, leaving a final sample of 807 cases. This sample was drawn from an ongoing clinical study conducted in an alcohol and drug outpatient setting. Connor et al. (18, forthcoming) and Young et al. (23) have used these data to validate cannabis expectancy and self-efficacy measures. Feeney et al. (25) examined the differences in mental health functioning between those who were and were not dependent on cannabis, as well as providing descriptive drug use data on 12 month and lifetime use. Human ethics approval was obtained from the Metro South Hospital and Health Service.

**Table 1:** **Past month alcohol and other drug use (*N* = 827)**.

	% used in past month	No. days used in past month	Average amount used per occasion
Alcohol	84.8	6.87 (SD = 8.46)	76.46 g (SD = 82.99)
Tobacco	64.8	27.31 (SD = 7.56)	14.13 (SD = 9.13)
Amphetamine	17.4	2.88 (SD = 4.32)	2.08 ‘points’ (SD = 2.41)
Ecstasy/MDMA	13.2	2.15 (SD = 2.36)	1.40 ‘tabs’ (SD = 1.15)
Heroin	4.5	8.32 (SD = 10.57)	3.87 g (SD = 11.02)
Benzodiazepines	4.2	15.09 (SD = 12.52)	16.88 mg (SD = 23.23)

*A ‘point’ is approximately 0.1 g*.

### Measures

#### Cannabis expectancy questionnaire

The Cannabis Expectancy Questionnaire (CEQ) is a 45-item questionnaire assessing positive (18 items, e.g., “I get better ideas when smoking cannabis”) and negative (27 items, e.g., “I am more worried about what others are saying about me when I am smoking cannabis”) cannabis use outcome expectancies (18, 26). There is a 5-point, Likert-style response format (1 = *Strongly Disagree* to 5 = *Strongly Agree*). The questionnaire was initially developed with a community sample and validated on a large sample of cannabis users recruited from a hospital outpatient clinic. The two subscales have high internal reliability (α ≥ 0.90), and the CEQ’s factor structure and criterion validity have been established across two samples (18).

#### Cannabis refusal self-efficacy questionnaire

The Cannabis Refusal Self-Efficacy Questionnaire (CRSEQ) is a 14-item questionnaire assessing an individual’s belief in their ability to resist smoking cannabis across various situations (23, 27). Items ask respondents to rate their ability to resist smoking cannabis on a 6-point Likert-type scale ranging from 1 (*I am very sure I could NOT resist smoking cannabis*) to 6 (*I am very sure I could resist smoking cannabis*). Similar to the Drinking Refusal Self-Efficacy Questionnaire [DRSEQ; (28)], it comprises three subscales: *Emotional Relief Self-Efficacy* (six items, e.g., “When I feel upset”), *Opportunistic Self-Efficacy* (five items, e.g., “When someone offers me a smoke”), and *Social Facilitation Self-Efficacy* (three items, e.g., “When I want to feel more confident”). The questionnaire was developed with a community sample and validated on a large sample of cannabis users recruited from an outpatient treatment service. The internal reliability is good/excellent (α = 0.84–0.97), and its factor structure and criterion validity has been previously established (23).

#### Severity of dependence scale-cannabis

The SDS-C is a 5-item screening questionnaire measuring the severity of cannabis dependence (29). The SDS-C is sensitive to severity of cannabis dependence (30). Using Australian normative data, the SDS-C cut-off for likely cannabis dependence is ≥3 (24).

#### General health questionnaire-28

The General Health Questionnaire-28 (GHQ-28) is a 28-item self-report measure which identifies short-term changes in health perception (31). It has four sub-scales (i) Somatic Symptoms, (ii) Anxiety, (iii) Social Dysfunction, and (iv) Depression (31). Higher sub-scale scores reflect poorer functioning. The GHQ-28 is a widely used measure of psychological health with strong psychometric properties (31–33).

#### Psychotic-like symptoms

Psychotic-like symptoms were assessed using the Positive Symptoms (five items) and Manic-Excitement (six items) sub-scales of the 24-item Brief Psychiatric Rating Scale [BPRS; (34)]. The BRPS is a clinician-rated scale measuring 24 different psychiatric symptoms, each rated on a 7-point scale, ranging from 1 (*not present*) to 7 (*extremely severe*). It is a reliable and valid measure of psychiatric symptoms (35), and has previously been administered to assess psychotic-like symptoms in injecting drug users (36). Masters- and PhD-qualified clinical psychologists administered the BPRS. Psychologists had between 2 and 25 years experience (*M* = 10.5 years).

#### Quantity and frequency

Quantity and frequency of alcohol and other drug use *in the past month* was assessed by Masters- and PhD-qualified clinical psychologists using a retrospective diary approach over the past month, past 12 months, and lifetime. As recommended by the State Health Service, to ensure consistent measurement of cannabis quantity across state-wide clinics ‘joints’ (cannabis cigarette) were quantified as 0.25 g of cannabis, and ‘cones’ (use of ‘bong’ or ‘pipe’), 0.10 g of cannabis.

### Analysis

Latent class analysis was performed to identify patterns of multiple drug use using last month use of seven drugs: cannabis, alcohol, amphetamine, heroin, benzodiazepine, ecstasy (MDMA), and tobacco. LCA is a technique that identifies sub-classes within a population based on similarity of response to measured variables (37). This technique is characterized by two sets of parameters: (1) The estimated proportion of each class in the population and (2) the probability of an individual in a particular class using a certain drug. Determination of the correct number of classes was based on the Bootstrap Likelihood Ratio Test (38) and Sample Size Adjusted Bayesian Information Criterion [SSABIC; (39)]. These two criteria have shown excellent performance in identifying the correct number of classes (40). In BLRT, a significant *p*-value indicates that a given model fits the data better than a model with one less class. For SSABIC, a lower value indicates better balance between model parsimony and model fit. In addition to these two criteria, the average posterior probabilities of class membership were used to evaluate classification quality. Average posterior probabilities close to one suggest clear classification. Model fitting began with a 1-class solution, and the number of classes was successively increased up to a 4-class solution. Once the optimal number of classes was determined, the profiles of participants in different classes were compared using ANOVA, Kruskal–Wallis, and χ^2^ test.

## Results

Model fit statistics for 1–4 class solutions are presented in Table 2. The 3-class solution had the lowest SSABIC and results from the BLRT indicate that it fitted the data significantly better than a 2-class solution, but not worse than the 4-class solution. In addition, the average posterior probabilities of class membership of a 3-class solution were over 0.90, which indicated clear classification. Therefore, it was selected as the optimal model.

**Table 2:** **Fit statistics of the unconditional latent class analysis**.

	Loglikelihood	BIC	SSABIC	BLRT *p*-value
1 Class	−2216.117	4479.087	4456.858	
2 Classes	−2168.662	4437.723	4390.090	<0.001
3 Classes	−2153.480	4460.907	4387.869	<0.001
4 Classes	−2145.417	4498.326	4399.883	0.21

**Figure 1:**
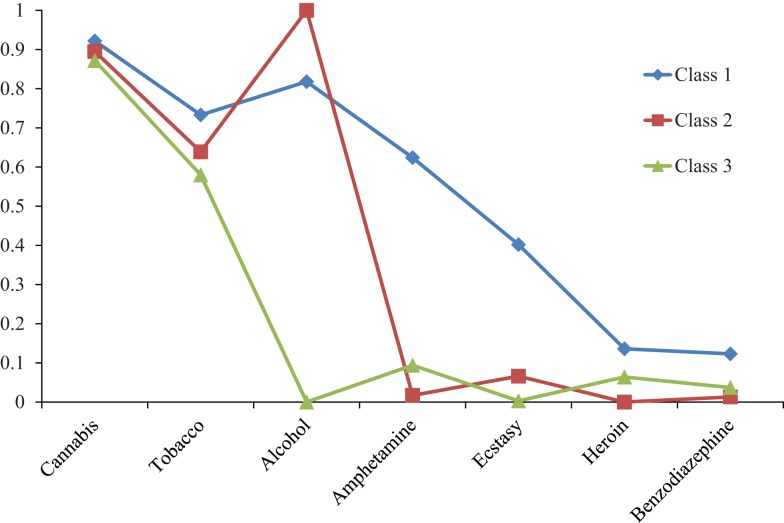
**Probability of last month substance use from the 3-class solution**.

Figure 1 shows the probability of last month use for each substance by class. Class 1 was characterized by wide-ranging substance use. Participants in this class had a high probability of cannabis, tobacco, alcohol, and amphetamine use, a moderate probability of ecstasy use, and a low probability of heroin and benzodiazepine use. This class was labeled as *wide-ranging substance use*, and the prevalence estimate of this class was 189 (23.5%). Class 2 was characterized by universal alcohol use, high probability of cannabis and tobacco use, and negligible probability of other drug use. This class was labeled as *cannabis, alcohol, and tobacco*, and the prevalence estimate of this class was 458 (56.8%). Class 3 was characterized by a high probability of cannabis and tobacco use, but negligible probability of other drug use. This class was labeled as *cannabis and tobacco*, and the prevalence estimate was 156 (19.8%).

Table 3 shows the profiles of the three classes. Participants in the *wide-ranging substance use* class had significantly higher negative cannabis expectancy, anxiety, and depression scores, lower emotional relief self-efficacy and lower social facilitation self-efficacy, and higher manic-excitement and positive psychotic symptoms (*p* < 0.05). They were also more likely to be cannabis dependent (*p* < 0.05). However, as shown in Table 3, the effect sizes were generally small, and in the case of psychotic-like symptoms, were very low in all groups. Cannabis users scored significantly higher than the Australian normative sample [Somatic Symptoms. 84, Anxiety. 77, Social Dysfunction. 64, Depression. 21; (41)] on all GHQ-28 subscales (*p*s < 0.05).

**Table 3:** **Profile of the three substance use classes**.

	Wide-ranging substance use			Cannabis, alcohol, and tobacco			Cannabis and tobacco			*F*	η^2^
	*N*	*M*	SD	*N*	*M*	SD	*N*	*M*	SD	
Age	132	24.92	6.45	498	25.16	8.61	176	26.79	8.77	2.85	0.007
**CANNABIS EXPECTANCY**											
Positive expectancy	120	49.85	11.44	450	49.78	10.71	153	50.07	11.78	0.04	0.001
Negative expectancy	117	69.63^a^	17.31	449	63.10^b^	16.38	147	61.83^b^	16.34	8.72***	0.024
**GHQ SUBSCALES**											
Somatic symptoms	131	1.23	1.68	499	0.91	1.44	175	0.84	1.41	3	0.008
Anxiety	131	1.60^a^	1.98	498	1.05^b^	1.69	175	1.10^b^	1.7	5.15**	0.013
Social dysfunction	131	0.87	1.41	496	0.7	1.41	172	0.84	1.52	1.11	0.003
Depression	131	0.85^a^	1.65	496	0.46^b^	1.28	172	0.60^ab^	1.55	3.94*	0.01
**CANNABIS REFUSAL SELF-EFFICACY**											
Emotional relief self-efficacy	120	21.93^a^	9.75	457	24.70^b^	9.01	149	23.39^ab^	9.13	4.73**	0.013
Opportunistic self-efficacy	121	15.63^a^	7.68	448	17.40^ab^	7.49	150	18.09^b^	7.5	3.83*	0.011
Social Facilitation self-efficacy	122	14.38	3.92	456	15.01	3.45	151	14.68	3.38	1.76	0.001
**BRIEF PSYCHIATRIC RATING SCALE**											
BPRS positive symptoms^†^	131	5.66^a^	1.35	499	5.48^b^	1.44	172	5.46^ab^	1.32	*p* = 0.022	
BPRS manic-excitement^†^	131	7.17^a^	1.91	498	6.69^b^	1.58	172	7.05^ab^	2.20	*p* = 0.007	

		***N***	**%**		***N***	**%**		***N***	**%**	**χ^2^**	**Cramer’s *V***

**GENDER**											
Male		103	78.03		394	79.12		125	71.02	4.9	0.078
Female		29	21.97		104	20.88		51	28.98		
**CANNABIS DEPENDENT**											
Dependent		76	57.58		213	42.77		85	48.3	9.52**	0.109
Not dependent		56	42.42		285	57.23		91	51.7		

*^ab^Means with the same superscript were not significantly different*.

*^†^Overall group difference tested using Kruskal–Wallis test. Follow-up pairwise comparisons tested using Mann–Whitney U tests with a Bonferroni-like adjustment to α (0.05/3 = 0.016)*.

*p < 0.05; **p < 0.01; ***p < 0.001.

## Discussion

This is the first study to examine the substance use profiles and co-existing mental health symptoms of individuals referred for cannabis use treatment, applying LCA. Previous LCA studies in cannabis users drawn from population and community samples typically assess lifetime or past 12 month use. To more precisely examine current polysubstance use, we restricted the timeframe to the past 4 weeks. LCA generated a three-class solution that included Class (1) *Wide-Ranging Substance Use*, Class (2) *Cannabis, Alcohol, and Tobacco Use*, and Class (3) *Cannabis and Tobacco Use*. As anticipated, prevalence rates of substance use were markedly higher than population-based studies. *Class 1* patients represented approximately one quarter of the sample. They reported a high probability of cannabis, tobacco, alcohol, and amphetamine use, as well as moderate ecstasy use and low heroin and benzodiazepine use in the previous month. *Class 1* also had significantly higher levels of cannabis dependence. Representing just over half of the sample, *Class 2* had high probabilities of alcohol, cannabis, and tobacco use, with limited other drug use. The final LCA solution (*Class 3*) consisting of approximately one fifth of the sample were characterized by low probability of alcohol and other drug use, but frequent cannabis and tobacco use. Given the shorter time period under investigation compared to population-based studies, this finding is particularly significant.

Across all three classes, mental health functioning of patients was significantly more impaired than community norms (41). Also consistent with our hypotheses, patients classified as Wide-Ranging Substance Users (Class 1) had significantly higher Depression and Anxiety scores than Classes 2 and 3. Wide-Ranging Substance Users also displayed significantly higher positive psychotic-like and manic symptoms compared to Class 2 (*Cannabis, Alcohol, and Tobacco Use*). However, given the low prevalence of such symptoms across all groups, this finding should be interpreted with some caution. These findings are similar to population-based LCAs that have measured mood and anxiety (3, 7), as well as more broadly defined psychological distress (8). This study provides additional evidence that cannabis users in treatment have a higher prevalence of poor co-morbid mental health functioning. Severity of mental health dysfunction increases when substances other than alcohol and tobacco are introduced.

In this young population of cannabis users, a clinically important finding was low probability of alcohol use in Class 3 (frequent cannabis and tobacco use only). Our original research hypotheses anticipated that patients who limited use of substances to cannabis only were expected to have more salient cannabis-related beliefs, and should have higher cannabis expectancy and lower cannabis refusal self-efficacy scores. In contrast, the Wide-Ranging Substance group (Class 1) reported significantly higher negative cannabis expectancies and lower emotional relief and opportunistic cannabis refusal self-efficacy beliefs, when compared to Class 2 and 3. In alcohol studies, consistent with SCT (14, 15), this combination of high expectancies and low self-efficacy has been associated with highest levels of consumption [e.g., (42–44)] and poorest treatment outcomes [e.g., (45)]. More recently, the combination of high expectancy/low self-efficacy has been demonstrated to be predictive of higher levels of cannabis dependence and cannabis consumption (Connor et al., forthcoming), placing wide-ranging substance users at elevated risk.

Given the psychometric similarity between expectancy and self-efficacy factors across drug classes (Connor et al., forthcoming), drug specific scales may be capturing additional risk of using multiple drugs. Support for this can be observed with generic, non-drug (general) self-efficacy being highly associated with substance use (46). Front line treatments for cannabis use disorders that hold strongest evidence for efficacy include Cognitive-Behavioral Therapy (CBT), MI, and Contingency Management (CM) (47). In the country this study was undertaken (Australia), CBT and MI are most widely used. Both expectancy and self-efficacy are key targets for CBT-based addiction treatments [e.g., (19, 22, 45)]. Cannabis users engaging in wide-ranging substance use may benefit from greater focus on enhancing strategies to cope with distress (for emotional relief self-efficacy, anxiety, depression) and general refusal skills (for opportunistic self-efficacy), and less on building motivation for change (negative expectancies).

The research has some limitations. The cross-sectional design does not allow interpretation of causality. Substance use was assessed though self-report. Biological verification would provide a more robust assessment of substance use. The design does not allow assessment of the specific role of cannabis versus other drugs in the severity of co-existing mental health problems, or cannabis-related cognitions. While the sample size for a clinical population is robust, the findings may not be generalizable to all treatment seeking populations. All patients attended under court direction as an alternative to a criminal prosecution. This may have had a proximal effect on self-reported health and functioning. Future work could assess patients over multiple time points to detect changes in substance use, mental health functioning, and cannabis-related beliefs. Prospective comparisons between patients formally engaged in cannabis treatment could assess the prognostic capacity of the three cannabis groups identified.

This LCA study in a group of cannabis users diverted to treatment identified that high levels of cannabis dependence and illicit polysubstance use were strongly associated with impaired mood and anxiety, as well as higher positive psychotic-like and manic symptoms. Treatment approaches for this more complex group may include combined CBT and pharmacotherapy to more effectively target these symptoms directly. Patients with wide-ranging substance use profiles may additionally benefit from psychologically based strategies that focus on more effectively coping with symptom distress. Based on findings that this higher risk profile has lower cannabis refusal self-efficacy beliefs, enhancing skills, and confidence to resist situational cues through behavioral training may provide additional clinical benefit in this higher risk group.

## Conflict of Interest Statement

The authors declare that the research was conducted in the absence of any commercial or financial relationships that could be construed as a potential conflict of interest.
